# Evolution of demarcation line after pneumatic retinopexy—a case report

**DOI:** 10.1186/s12886-023-03184-w

**Published:** 2023-10-27

**Authors:** Ramesh Venkatesh, Vishma Prabhu, Ashit Handa, Isha Acharya, Rubble Mangla, Aishwarya Joshi, Jay Chhablani

**Affiliations:** 1 Dept. of Retina and Vitreous, Narayana Nethralaya, #121/C, 1st R Block, Chord Road, Rajaji Nagar, 560010 Bengaluru, Karnataka India; 2 Medical Retina and Vitreoretinal Surgery, University of Pittsburgh School of Medicine, 203 Lothrop Street, Suite 800, 15213 Pittsburg, PA USA

**Keywords:** Rhegmatogenous retinal detachment, Demarcation line, Pneumatic retinopexy, Position

## Abstract

**Background:**

Demarcation line in a rhegmatogenous retinal detachment (RD) is a classic finding noted in chronic cases. In this case report, we describe a case of evolution of post-operative demarcation line after pneumatic retinopexy (PnR) in a subtotal rhegmatogenous RD.

**Case description:**

A 31-year-old male diagnosed with acute, subtotal, macula-off rhegmatogenous RD in the left eye of 15-day duration underwent PnR on the same day. His presenting visual acuity was 6/48 in the left eye. Transconjunctival cryopexy was performed to the retinal break at the same sitting and 0.5 cc of 100% perfluoroproprane (C3F8) gas was injected in the vitreous cavity and right lateral position was advised to the patient.

**Result:**

A pigmentary demarcation line was noted extending the nasal part of the macula along the most dependent part of the detachment on the immediate post-operative day and was more obviously visible on the 2nd and then on the 11th post-operative day. The visual acuity at the last follow-up visit improved to 6/18. Successful reattachment of the retina was noted on the last follow-up visit.

**Conclusion:**

Post-operative demarcation lines after RD surgery could develop due to subretinal migration of pigments and along the most-dependent part depending upon post-operative positioning of the patient. Careful post-operative positioning, particularly in macula splitting RDs could be important to avoid pigment accumulation along the foveal area.

## Background

A rhegmatogenous retinal detachment (RD) is a type of RD caused by a retinal tear of full thickness that allows vitreous fluid to enter the subretinal space [1]. In rhegmatogenous RD, the retinal pigment epithelial cells remain continually exposed to the subretinal space and/or vitreous cavity, resulting in the development of proliferative vitreoretinopathy [2]. Chronic rhegmatogenous RD is characterized by demarcation lines, which are linear or curved lines concentric around the retinal break. They are caused by the proliferation of retinal pigment epithelial cells at the juncture of the attached and detached retina and limit the progression of RD as a result [3].

Pneumatic retinopexy (PnR) is a non-incisional, minimally invasive technique for repairing RD [4]. It involves three essential steps: (a) the injection of an expandable gas, (b) the application of retinal cryotherapy or laser photocoagulation to seal retinal breaks, and (c) the maintenance of an appropriate head position. The prescribed head position is determined by the location of the primary retinal break, so that the retinal break occupies the 12 o’clock meridian and the RD is most dependent on the macular region [5]. It is an important tool in the vitreoretinal surgeon’s armamentarium, yielding positive results in carefully selected patients where pars plana vitrectomy is not available or cannot be performed.

This report describes a case of subtotal acute RRD in which a demarcation line appeared following a successful PnR, and the plausible causes for its development.

## Case description

**Fig. 1:**
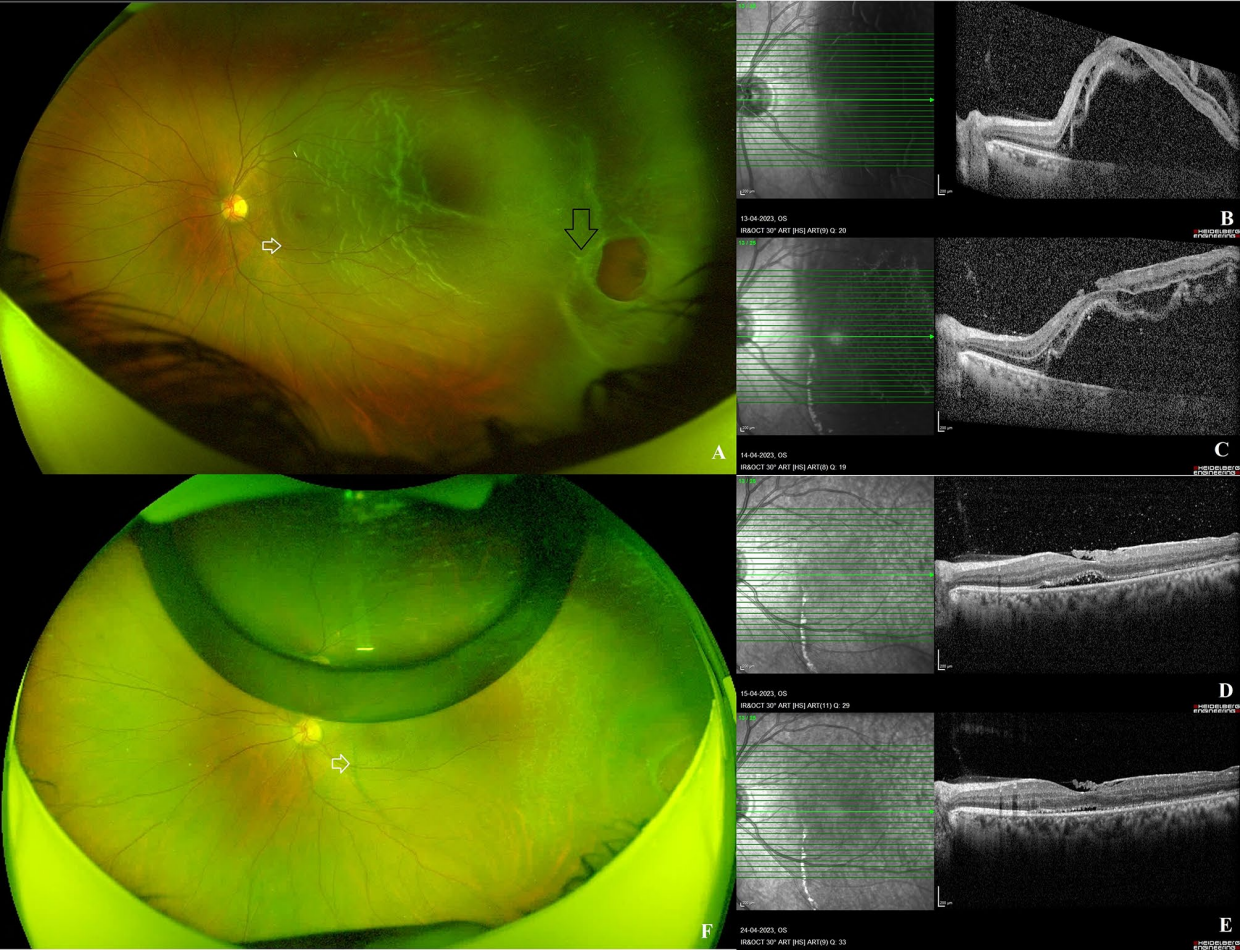
Clinical and imaging features at presentation and at last follow-up visit: **A**: Left eye fundus photograph (Optos®, Daytona, USA) demonstrates a subtotal temporal macula-off retinal detachment with retinal corrugations and a large oval retinal break at the temporal retinal periphery with rolled-up posterior margins (black arrow). There was no demarcation line identified (white arrow). **B**-**E**: Optical coherence tomography (OCT) scans passing through the macula on the same day before the pneumatic retinopexy procedure and on days 1, 2, and 11 after the procedure reveal a progressive decrease in subretinal fluid and retinal reattachment. The infrared images on the OCT scans demonstrate the formation of the demarcation line as a hyperreflective white band extending from the nasal aspect of the fovea to the inferior arcade. **F**: This postoperative fundus image obtained at the most recent follow-up appointment demonstrates retinal reattachment and clearly identifies the demarcation line at the junction of the attached and previously detached retina (white arrow)

A 31-year-old male presented to the retinal clinic of a tertiary eye hospital with complaints of sudden loss of vision in the left eye for the last 15 days. He had no history of ocular trauma or any other systemic illness. The present visual acuity of the right eye was 6/6 and the left eye was 6/48, respectively with emmetropic refraction. Examination of the anterior segment and measurement of intraocular pressure in both eyes were normal. A dilated fundus examination of the left eye revealed a clear media, normal retinal vasculature, and a subtotal temporal macula-off RD with retinal corrugations. A large oval retinal break was identified on the temporal aspect of the peripheral retina, anterior to the equator at the 4 o’clock meridian, measuring nearly two-disc diameters laterally and having rolled-up posterior margins. There were no indications of chronic RD, such as demarcation line, retinal cyst, subretinal or preretinal membranes (Fig. 1A). Horizontal optical coherence tomography scan obtained with Spectralis machine (Heidelberg Engineering, Germany) revealed detached retina extending up to 1-disc diameter nasal to the fovea. On the optical coherence tomography scan, outer retinal corrugations were detected. The underlying retinal pigment epithelium appeared normal throughout the entire scan (Fig. 1B). After a thorough discussion with the patient regarding the various treatment options, the decision was made to perform a single-step PnR with same sitting transconjunctival cryotherapy on the same day in a sterile operating room.

Under direct observation with an indirect ophthalmoscope, a single freeze-thaw technique of transconjunctival cryotherapy was performed around the retinal break during the procedure. Multiple cryo spots were applied. During cryotherapy, care was taken to avoid repeatedly treating the same area and directly under the retinal tear. After performing anterior chamber paracentesis and achieving adequate hypotony, 0.5 cc of 100% perfluoropropane (C3F8) gas was injected into the vitreous cavity. By repeatedly tapping the eyeball with a cotton applicator, multiple small gas bubbles coalesced into one large gas bubble. The intraocular pressure was measured digitally, and perfusion of the optic disc was observed with an indirect ophthalmoscope. Patient was instructed to assume a right-lateral position for at least 5 days after eye patching.

**Fig. 2:**
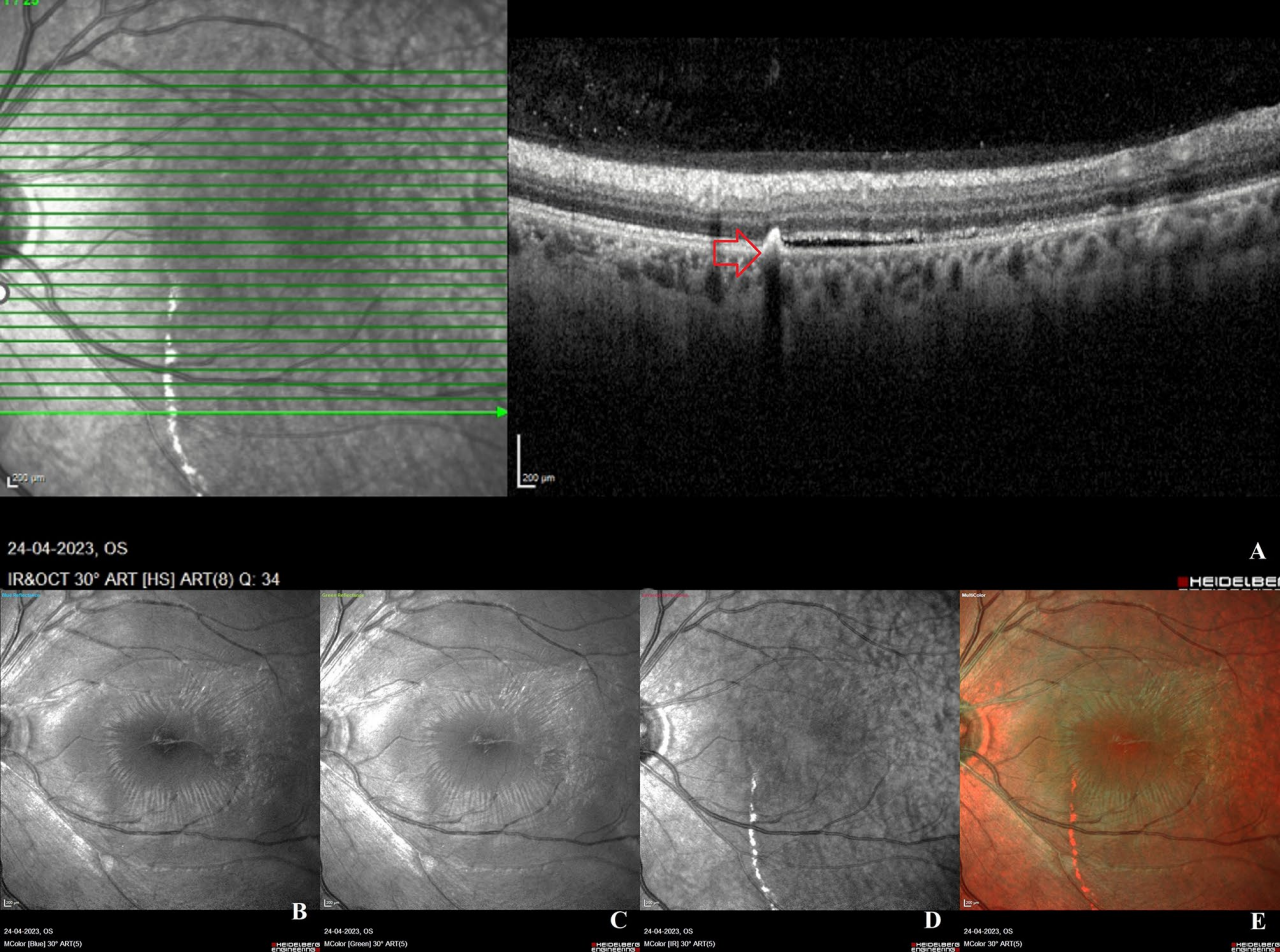
Optical coherence tomography (OCT) through the demarcation line and Multicolour® imaging of the left eye on the final post-operative visit: **A**: On OCT, a small clump of white hyperreflective material with underlying shadowing is observed in the subretinal space at the exact junction of the attached and detached retina. **B**-**E**: The demarcation line is not discernible on the individual blue and green reflectance channels. On the infrared reflectance channel, the demarcation line appears as a thick, hyperreflective, white line that extends from the fovea to the inferior retinal arcades. In the composite Multicolour® image, the demarcation line appears orange

The anterior segment examination and intraocular pressure measurement of the left eye on the first postoperative day were within normal limits. Left fundus dilation revealed a single gas bubble with decreased subretinal fluid. In addition, a faintly visible vertically oriented black pigmentary line was observed at the junction of the attached and detached retina on the nasal aspect of the fovea extending beyond the inferior retinal arcade during the clinical examination, along the most dependent part of the RD. The patient was instructed to maintain the post-operative position and return the next day for a follow-up. The following day, the left eye’s visual acuity had improved to 6/18 and the subretinal fluid had significantly decreased. At this visit, the pigmentary line was prominent. At the final visit 11 days after the procedure, the RD had completely reattached, and the pigmentary line was now clearly visible (Fig. 1F). At each follow-up appointment, optical coherence tomography scans revealed the regression of subretinal fluid and subsequent retinal reattachment (Fig. 1C-E). A small clump of white hyperreflective material with underlying shadowing was observed in the subretinal space precisely at the interface between the attached and previously detached retina (Fig. 2A). On the Spectralis, Multicolour® imaging of the posterior pole was performed. The blue and green reflectance channels did not show the demarcation line, whereas the infrared reflectance channel revealed the pigmentary line to be a thick, discontinuous, hyperreflective white line that extended beyond the inferior retinal arcade (Fig. 2B-E).

## Discussion and conclusion

This report describes a patient with subtotal acute RD who developed a pigmented demarcation line following a successful PnR procedure to reattach the retina along the most dependent part of the RD while maintaining post-op positioning. The location of the retinal break (single, temporal break within one clock hour), the characteristics of the RD (recent onset subtotal RD with minimal proliferative vitreoretinopathy changes) and the patient characteristics (young patient with no preexisting glaucoma history and with no problem in maintaining the required post-procedure position) made this an ideal case for PnR. The potential reasons cause for the development of the post-operative demarcation line in this case is provided herewith. The infrared channel’s hyper reflectance confirms the presence of melanin and, by extension, retinal pigment epithelial cells in the subretinal space. Melanin absorbs light with short wavelengths and reflects light with longer wavelengths [6, 7]. The excessive release of retinal pigment epithelial cells into the subretinal space and/or vitreous cavity can occur during cryopexy of the retinal break [8]. Frequent tapping of the eyeball to form a single gas bubble during retinopexy may also result in a large quantity of retinal pigment epithelial cell release. The persistent traction at the retinal break’s posterior margin would facilitate the release of retinal pigment epithelial cells into the subretinal space. In addition, the patient’s position in our case would make the junction of the attached and detached retina the most dependent site, allowing the accumulation of retinal pigment epithelial cells to lead to the formation of a demarcation line.

Following the procedure, visual acuity improved in this case as the demarcation line was away from the foveal center. The accumulation of retinal pigment epithelial cells in the foveal neurosensory space could result in diminished visual acuity. To prevent the accumulation of retinal pigment epithelial cells at the fovea, it would be necessary to take certain measures when performing PnR. These include a PnR in which only a small gas bubble is initially injected, followed by retinopexy with not-so-heavy laser burns or cryotherapy around the retinal break as soon as the retina attaches to prevent the release of retinal pigment epithelial cells and close the subretinal space’s entrance [4]. Avoid multiple ‘fish-egg’ gas bubbles and achieve a single large bubble by injecting briskly within the bubble [4]. Using the steam roller positioning technique in PnR would permit early reattachment of the macula, thereby preventing retinal pigment epithelial cells from gaining access to the neurosensory space at the fovea, particularly in cases of macula-off RDs [5, 9]. Pars plana vitrectomy could also be used to obtain a more rapid macular attachment, particularly in eyes that are not feasible to PnR, so as to avoid the development of the foveal demarcation line.

In conclusion, post-operative demarcation lines can develop after PnR along the most dependent part of the RD during post-operative positioning. Steps taken to prevent pigments from entering the subretinal space will prevent this potentially vision-threatening complication.

## Data Availability

The datasets used and/or analysed during the current study are available from the corresponding author on reasonable request.
